# Lifestyle and factors of vascular and metabolic health and inflammation are associated with sensorineural-neurocognitive aging in older adults

**DOI:** 10.3389/fepid.2023.1299587

**Published:** 2024-01-05

**Authors:** Natascha Merten, Mary E. Fischer, Aaron Alex Pinto, Richard J. Chappell, Carla R. Schubert

**Affiliations:** ^1^ Department of Population Health Sciences, School of Medicine and Public Health, University of Wisconsin-Madison, Madison, WI, United States; ^2^ Division of Geriatrics and Gerontology, Department of Medicine, School of Medicine and Public Health, University of Wisconsin-Madison, Madison, WI, United States; ^3^ Wisconsin Alzheimer’s Disease Research Center, School of Medicine and Public Health, University of Wisconsin-Madison, Madison, WI, United States; ^4^ Department of Ophthalmology and Visual Sciences, School of Medicine and Public Health, University of Wisconsin-Madison, Madison, WI, United States; ^5^ Department of Biostatistics and Medical Informatics, School of Medicine and Public Health, University of Wisconsin-Madison, Madison, WI, United States

**Keywords:** hearing, olfaction, cognition, neuronal function, brain aging, longitudinal

## Abstract

This study's aim was to identify risk factors associated with sensorineural and neurocognitive function (brain aging) in older adults. In *N* = 1,478 Epidemiology of Hearing Loss Study participants (aged 64–100 years, 59% women), we conducted sensorineural and cognitive tests, which were combined into a summary measure using Principal Component Analysis (PCA). Participants with a PCA score <−1 standard deviation (SD) were considered to have brain aging. Incident brain aging was defined as PCA score <−1 SD at 5-year follow-up among participants who had a PCA score ≥−1 SD at baseline. Logistic regression and Poisson models were used to estimate associations between baseline risk factors of lifestyle, vascular and metabolic health, and inflammation and prevalent or incident brain aging, respectively. In an age-sex adjusted multivariable model, not consuming alcohol (odds ratio(OR) = 1.77, 95% confidence Interval (CI) = 1.18,2.66), higher interleukin-6 levels (OR = 1.30, 95% CI = 1.03,1.64), and depressive symptoms (OR = 2.44, 95% CI = 1.63,3.67) were associated with a higher odds of having brain aging, while higher education had protective effects (OR = 0.55, 95% CI = 0.33,0.94). A history of stroke, arterial stiffness, and obesity were associated with an increased risk of developing brain aging during the five years of follow-up. Lifestyle, vascular, metabolic and inflammatory factors were associated with brain aging in older adults, which adds to the evidence of shared pathways for sensorineural and neurocognitive declines in aging. Targeting these shared central processing etiological factors with interventions may lead to retention of better neurological function, benefiting multiple systems, i.e., hearing, smell, and cognition, ultimately helping older adults retain independence and higher quality of life longer.

## Introduction

Many studies have shown that age is a major risk factor for the development of sensorineural impairments as well as cognitive impairments (1–4). In our aging (Western) populations, sensory and cognitive impairments and dementia are thus becoming a growing public health concern, particularly, given the lack of effective prevention and treatment methods.

Sensory and cognitive impairments often co-occur in aging adults and sensory impairments have been associated with cognitive impairment and dementia (5–12). Previous epidemiological studies have also reported associations of vascular, metabolic, and inflammatory factors with both sensorineural and with neurocognitive declines and impairments and dementia (2, 5, 7, 13–27). Studied risk factors include education, smoking, alcohol consumption, weight, physical activity, and chronic health conditions. Importantly, some of these health and lifestyle factors are considered modifiable and could, thus, present an opportunity to inform future intervention and prevention strategies for neurodegeneration and brain aging.

Vascular, metabolic and inflammatory risk factors and their downstream pathological processes may contribute to systemic neurodegeneration that affects the brain, including the processing areas for cognitive functions as well as those for sensorineural systems. These shared etiologic pathways may thus be one of the underlying reasons for the observed relationships between sensorineural and neurocognitive functions. Previous studies on sensory and neural function primarily studied the associations of senses as a determinant and cognition as an outcome (5, 7–12). Moreover, previous studies on vascular, metabolic, and inflammatory risk factors have often been limited to individual sensory or cognitive outcomes (2, 5, 7, 13–17, 19–27). Although the idea of a common cause for generalized underlying neurodegeneration has been receiving more attention over the past years (6, 28, 29), the study of a composite sensorineural-neurocognitive function remains scarce. In previous work in the Beaver Dam Offspring Study (BOSS), a summary measure combining function on sensorineural and neurocognitive tests as a measure of brain aging has been established (18). This study found that vascular-related risk factors and markers of inflammation were associated with brain aging (18) in a cohort of primarily middle-aged adults. Moreover, our work from another previous study showed that a measure of retinal thickness, which is considered a potential early marker for preclinical decline within the central nervous system and its functionality, was thinner in people with more brain aging (29).

While this study was conducted in generally rather healthy middle-aged adults, less is known about sensorineural-neurocognitive (brain) aging in older adults, who may be further along the pathology spectrum. Thus, the aim of this study was to determine the association of risk factors of lifestyle, vascular and metabolic health and inflammation with sensorineural-neurocognitive (brain) aging in older adults.

## Methods

### Study population

The Epidemiology of Hearing Loss Study (EHLS) is a longitudinal, population-based study of sensory and cognitive aging (23, 30–32). Baseline Beaver Dam Eye Study (1987–1988) participants were eligible for participation in EHLS. The baseline EHLS examination was conducted 1993–1995 (participants aged 48–92 years), with follow-up examinations every 5 years (1998–2000; 2003–2005; 2009–2010; 2013–2015) (30, 33). Similar standardized protocols were used at each study phase. Hearing testing was conducted at all phases and olfactory and cognitive testing began at the 2003–2005 follow-up (34, 35). The baseline for the current study was the 15-year follow-up when an extensive cognitive battery was included (20, 22). Data from the 20-year follow-up were used for 5-year incidence analyses. All study phases were approved by the Health Sciences Institutional Review Board of the University of Wisconsin and written informed consent was obtained prior to each examination.

### Measurements

#### Sensorineural function

Audiometric testing was performed following the American Speech-Language-Hearing Association guidelines and was in compliance with American National Standards Institute (ANSI) standards (36, 37). The pure-tone average (PTA) of thresholds at 0.5, 1, 2, and 4 kHz of the worse ear was used as the measure of hearing function (30, 32).

Olfactory function was measured with the eight-item San Diego Odor Identification Test (SDOIT) (34). Odorants were presented in a random order with a minimum of 45 s between odors. A picture board that included the eight odorants plus 12 distractor items was available to aid identification. The score was the total number of correctly identified odors (23, 34, 38).

#### Neurocognitive function

A cognitive function tests battery was administered at the 15- and 20-year follow-up, measuring cognitive dimensions of executive function, attention, speed, memory and language (Trail-Making Test A and B (TMTA, TMTB), Digit Symbol Substitution Test (DSST), Rey Auditory Verbal Learning Test (AVLT), Verbal Fluency Test (VFT) and Mini-Mental State Examination (MMSE)) (20, 22, 39).

As part of the health history interview, memory problems and previous diagnoses of Alzheimer's Disease or dementia were recorded (20).

#### Risk factors of lifestyle, vascular and metabolic health and inflammation

Risk factors were obtained at the 15-year examination. Some data were collected as part of the Beaver Dam Eye Study, a concurrent study in the same population (33). Blood pressure was measured and hypertension was defined as a systolic blood pressure ≥140 mmHg or diastolic blood pressure ≥90 mmHg or a diagnosis of high blood pressure with current anti-hypertensive medication use. Height and weight were measured, and body mass index was calculated as kilograms per square meter. Obesity was defined as BMI > 30 kg/m^2^. High resolution B-mode carotid artery ultrasound images were obtained (MyLab25, Esaote North America, Indianapolis, IN) and carotid intima-media thicknesses (IMT) of the near and far walls of the common carotid, bifurcation, and internal carotid artery on the left and right sides were measured (22). Mean IMT was the average thickness of the 12 walls. Plaque was indicated by acoustic shadowing with a change in the wall shape, texture or an IMT > 1.5 mm or, in the absence of acoustic shadowing, at least two of the other characteristics were present (22). Carotid femoral pulse-wave velocity was measured using the Complior SP system (Alam Medical, Saint-Quentin-Fallavier, France) and arterial stiffness was defined as a pulse-wave velocity greater than 12 m/s (40).

Non-fasting blood samples were analyzed for Hemoglobin A1C (HbA1C; Tosoh HPLC G7 Glycohemoglobin Analyzer, Tosoh Medics, Inc., San Francisco CA), total and high density lipoprotein (HDL) cholesterol (Roche Modular P Chemistry Analyzer, Roche Diagnostics Corporation, Indianapolis, IN), high sensitivity C-reactive protein [hsCRP; latex particle-enhanced immunoturbidimetric assay kit, Roche Diagnostics, Indianapolis, IN; interassay coefficient of variation (CV) = 4.5%] and interleukin-6 (IL-6; QuantiKine High Sensitivity kit, R & D Systems, Minneapolis, MN; CV = 11.7%) (17). Diabetes was defined as HbA1C ≥ 6.5% or a diagnosis of diabetes or suspected diabetes with current treatment. Non-HDL cholesterol was total minus HDL cholesterol. Inflammatory markers were log transformed [natural log (ln)] and analyzed as continuous variables.

Interviewer-administered questionnaires obtained information on demographic (age, sex, education) and behavioral factors (smoking, alcohol, exercise, head injury), and vascular health-related history, including a history of stroke (24, 41). CVD was considered present if the participant reported a history of any one of eleven physician-diagnosed conditions or procedures (myocardial infarction, angina, stroke, transient ischemic attack, congestive heart failure, peripheral vascular disease, deep vein thrombosis, angioplasty, bypass, carotid artery surgery, or surgery to restore blood flow to the legs). The Centers for Epidemiological Studies Depression Scale (CES-D) was used to evaluate the presence of depressive symptoms (score >15) (42).

All measures were obtained by trained and certified study examiners.

### Statistical methods

All analyses were completed using the using SAS version 9.4 (SAS Institute, Inc. Cary, NC).

#### Calculation of brain aging

To represent the integrity of the entire sensorineural system, we calculated a combined sensorineural-neurocognitive function (brain aging) score, as a composite of sensorineural (PTA, SDOIT) and neurocognitive (TMTA, TMTB, DSST, AVLT, VFT, MMSE) function test data using principal component analysis (PCA) with the factor procedure (43). This concept and approach have been previously published (18). We used the measures of the EHLS 15-year examination, which was the baseline for this study. We inverted scores of PTA, TMTA, and TMTB so that for all measures a higher score indicated better function. We applied the eigenvalue greater than 1 rule and retained the first component (44). Each participant's PCA score represented a composite measure of their performance on the sensorineural and neurocognitive function tests relative to the study population. The scores had a mean of zero and a standard deviation (SD) of 1. A higher score indicated better overall performance. Participants with a PCA score more than 1 SD below the mean (<−1) were classified as having prevalent brain aging. For more details on the PCA see Supplementary Material S1.

To evaluate factors associated with the 5-year incidence of brain aging, a PCA score was calculated using sensorineural and neurocognitive data from the EHLS 20-year examination using the same methods and factor loadings. Incident brain aging was defined as a PCA score more than 1 SD below the mean (<−1) at the 20-year examination among participants who had a PCA score ≥−1 at the 15-year examination.

In this older population, some participants were unable to complete all of the tests required to be included in the PCA. In secondary analyses, we classified participants with mild cognitive impairment (MCI), cognitive impairment or dementia (20) who were not included in the PCA because of incomplete data at the baseline, or follow-up, as having prevalent or incident brain aging, respectively.

#### Statistical analyses

We used logistic regression models to determine the association of each individual baseline risk factor with prevalent brain aging, generating odds ratios (OR) and 95% confidence intervals (CI). To determine associations of baseline risk factors with 5-year incidence in brain aging, we estimated the relative risk (RR) and 95% CI by fitting Poisson models to a binary response via Proc Genmod. All models were adjusted for age and sex and repeated in sex-specific models adjusted for age. Factors that were statistically significant (*p* < 0.05) in individual age-sex adjusted models were then tested in multivariable models. A manual stepwise selection was used to construct multivariable models. To reduce the potential effects of collinearity, similar covariates (e.g., IMT and plaque) were tested separately in models.

This process was repeated in the secondary analyses. Factors were retained in the final multivariable models if they were significant in either the main or secondary analyses.

## Results

Of the *N* = 1,857 participants at the 15-year follow-up phase, *N* = 1,478 (80%) participants had complete sensorineural and neurocognitive data and were included in the PCA. Participants had a mean age of 75 years (range: 64–100 years), and 59% were women. At baseline, *N* = 208 participants had a PCA score less than −1 and were thus classified as having brain aging. There were *N* = 135 participants with MCI, cognitive impairment, or dementia without a PCA score who were added to the brain aging group for secondary analyses. Participants with brain aging at baseline were more likely to be men (OR = 2.59, 95% CI = 1.84, 3.64, adjusted for age) and to be older (OR = 2.40, 95% CI = 2.11, 2.72, per 5-year increase, adjusted for sex). More study cohort details can be found in Table 1 and Supplementary Material S2.

**Table 1:** Baseline characteristics and age-sex adjusted associations of risk factors with prevalent brain aging in the EHLS.

Participant characteristic	Prevalent brain aging			
	*N*	Yes *N* (%)	No *N* (%)	OR (95% CI)^a^
Sex, male	1,478	110 (52.9)	510 (40.2)	2.59 (1.84, 3.64)^b^
Education 16 + years	1,444	26 (12.9)	276 (22.2)	0.45 (0.27, 0.73)
Current smoker	1,478	12 (5.8)	104 (8.2)	1.02 (0.52, 2.01)
No alcohol past year	1,472	72 (34.8)	288 (22.8)	2.07 (1.43, 2.98)
History heavy alcohol use	1,475	65 (31.3)	297 (23.4)	1.68 (1.13, 2.50)
Exercise, ≥1/week	1,475	61 (29.5)	609 (48.0)	0.71 (0.49, 1.01)
Obesity (BMI > 30 kg/m^2^)	1,426	91 (46.7)	662 (53.8)	0.99 (0.70, 1.40)
Head injury	1,475	82 (39.4)	519 (41.0)	0.82 (0.59, 1.15)
Depressive symptoms	1,417	71 (38.0)	186 (15.1)	2.69 (1.83, 3.96)
Cardiovascular disease	1,466	87 (43.3)	361 (28.5)	1.11 (0.78, 1.57)
Stroke	1,472	12 (5.9)	28 (2.2)	2.12 (0.97, 4.62)
Hypertension	1,466	144 (71.6)	846 (66.9)	1.13 (0.78, 1.63)
Diabetes	1,478	51 (24.5)	275 (21.7)	1.12 (0.76, 1.64)
Hemoglobin A1C ≥ 6.5%	1,443	34 (16.8)	172 (13.9)	1.36 (0.87, 2.13)
Carotid artery plaque >2	1,362	108 (62.8)	487 (40.9)	1.31 (0.91, 1.90)
Arterial stiffness	1,287	71 (44.1)	319 (28.3)	0.94 (0.64, 1.38)
		Mean (SD)	Mean (SD)	
Age (per + 5 years)	1,478	81.4 (7.4)	73.5 (6.2)	2.40 (2.11, 2.72)^c^
Mean IMT (per + 0.1 mm)	1,432	1.00 (0.30)	0.87 (0.24)	1.04 (0.97, 1.11)
Non-HDL cholesterol (per + 30 mg/dl)	1,445	133.3 (39.1)	139.6 (38.5)	0.99 (0.86, 1.13)
HDL cholesterol (per + 10 mg/dl)	1,445	50.5 (15.1)	54.1 (16.1)	0.88 (0.78, 0.995)
hsCRP (mg/L)	1,414	5.1 (15.4)	3.6 (6.8)	1.06 (0.90, 1.23)^d^
IL-6 (pg/ml)	1,414	3.2 (3.8)	2.5 (4.3)	1.37 (1.11, 1.68)^d^

Prevalent Brain Aging was defined as having a principal component analysis (PCA) score <−1; there were *n* = 208 with prevalent brain aging and *n* = 1,270 without brain aging at this study's baseline (EHLS 15-year follow-up: *n* = 1,478); EHLS, epidemiology of hearing loss study; BMI, body mass index; OR, odds ratio; CI, confidence interval; HDL, high density lipoprotein; hsCRP, high sensitivity C-reactive protein; IL-6, interleukin-6; IMT, carotid artery intima media thickness.

^a^ Results of logistic regression models with each individual baseline risk factor as determinant and prevalent brain aging as outcome adjusted for age and sex.

^b^ Adjusted for age only.

^c^ Adjusted for sex only.

^d^ Natural log transformed (ln).

### Risk factors for prevalent brain aging

In age-sex-adjusted models, we found that less years of education, no alcohol in the past year, a history of heavy drinking, the presence of depressive symptoms, and elevated IL-6 and lower HDL cholesterol were associated with increased odds of brain aging (Table 1). In addition to these factors, a history of stroke (OR = 2.66, 95% CI = 1.36, 5.20), HbA1C ≥ 6.5% (OR = 1.62, 95% CI = 1.09, 2.40), weekly exercise (OR = 0.67, 95% CI = 0.49, 0.92), and carotid artery IMT (OR = 1.07, 95% CI = 1.004, 1.13, per 0.1 mm increase) and plaque (OR = 1.45, 95% CI = 1.04, 2.03, >2 sites) were also associated with brain aging in the secondary analyses including those with cognitive impairment/dementia. Results were similar in sex-specific analyses.

In a multivariable model including age, sex (OR = 2.88, 95% CI = 1.88, 4.43, men), education (OR = 0.55, 95% CI = 0.33, 0.94, 16 + years), no alcohol in the past year (OR = 1.77, 95% CI = 1.18, 2.66), depressive symptoms (OR = 2.44, 95% CI = 1.63, 3.67) and elevated IL-6 (OR =  1.30, 95% CI = 1.03, 1.64) remained associated with brain aging (Table 2, Model 1). A history of heavy drinking (OR = 1.66, 95% CI = 1.10, 2.50) was also significant in the secondary analyses (Table 2, Model 2). Stroke was not statistically significant in the final multivariable models but was retained in the models due to the large effect size in the secondary analyses. HDL, HbA1C, carotid IMT and plaque and exercise were not retained in the multivariable models.

**Table 2:** Multivariable models of risk factors and prevalent brain aging.

Baseline risk factors	Model 1^a^	Model 2^b^
	OR (95% CI)	OR (95% CI)
Age (per + 5 years)	2.40 (2.08, 2.77)	2.35 (2.06, 2.68)
Sex (Male)	2.88 (1.88, 4.43)	2.40 (1.62, 3.56)
Education (≥16 years)	0.55 (0.33, 0.94)	0.56 (0.35, 0.91)
No alcohol past year	1.77 (1.18, 2.66)	1.79 (1.23, 2.60)
History heavy alcohol use	1.46 (0.94, 2.29)	1.66 (1.10, 2.50)
Depressive symptoms	2.44 (1.63, 3.67)	2.51 (1.73, 3.66)
Stroke	1.59 (0.65, 3.91)	2.10 (0.96, 4.62)
Ln IL-6	1.30 (1.03, 1.64)	1.39 (1.12, 1.73)

CI, confidence interval; OR, odds ratio; Ln IL-6, natural log of interleukin-6.

^a^ Results of the multivariable logistic regression model with baseline risk factors as determinants and prevalent brain aging as outcome; *N* = 1,350’.

^b^ Results of the multivariable logistic regression model with baseline risk factor as determinants and prevalent brain aging as outcome, including participants with cognitive impairment/dementia without a PCA score in the brain aging group; *N* = 1,398 (secondary analyses).

### Risk factors for 5-year incidence of brain aging

There were *N* = 822 participants without brain aging or cognitive impairment at baseline with complete sensorineural and neurocognitive test data at follow-up that were included in the PCA for the analyses of 5-year incident brain aging; *N* = 73 participants were classified as having incident brain aging. An additional *N* = 26 participants with cognitive impairment/dementia were included in the brain aging group in secondary analyses. Being older (RR = 2.01, 95% CI = 1.76, 2.29, per 5-year increase, adjusted for sex) or a man (RR = 1.78, 95% CI = 1.17, 2.71, adjusted for age) was associated with an increased risk for brain aging (Table 3).

**Table 3:** Age-sex adjusted associations of risk factors with 5-year incident brain aging.

Participant characteristic	5-year incident brain aging			
	*N*	Yes *N* (%)	No *N* (%)	RR (95% CI)^a^
Sex (Male)	822	34 (46.6)	290 (38.7)	1.78 (1.17, 2.71)^b^
Education (16 + years)	809	12 (16.7)	187 (25.4)	0.64 (0.35, 1.14)
Current Smoker	822	2 (2.7)	58 (7.7)	0.53 (0.14, 2.04)
No alcohol past year	822	13 (17.8)	142 (19.1)	0.94 (0.52, 1.70)
History heavy alcohol use	822	20 (27.4)	162 (21.6)	1.48 (0.87, 2.50)
Exercise, ≥1/week	822	32 (43.8)	394 (52.6)	0.98 (0.63, 1.53)
Obesity (BMI > 30 kg/m^2^)	810	44 (60.3)	403 (54.7)	1.57 (1.02, 2.42)
Head injury	820	31 (43.1)	299 (40.0)	0.98 (0.63, 1.51)
Depressive symptoms	807	18 (25.4)	89 (12.1)	1.68 (1.02, 2.78)
Cardiovascular disease	820	28 (39.4)	198 (26.4)	1.14 (0.73, 1.79)
Stroke	822	6 (8.2)	11 (1.5)	3.43 (1.98, 5.95)
Hypertension	819	55 (75.3)	482 (64.6)	1.39 (0.85, 2.27)
Diabetes	822	22 (30.1)	148 (19.8)	1.49 (0.95, 2.36)
Hemoglobin A1C ≥ 6.5%	812	13 (18.1)	98 (13.2)	1.28 (0.75, 2.19)
Carotid artery plaque >2	780	33 (47.8)	255 (35.9)	0.96 (0.60, 1.54)
Arterial stiffness	742	27 (40.9)	152 (22.5)	1.23 (0.76, 1.98)
		Mean (SD)	Mean (SD)	
Age (per + 5 years)	822	78.1 (5.6)	71.9 (5.50)	2.01 (1.76, 2.29)^c^
Mean IMT (per + 0.1 mm)	815	0.92 (0.24)	0.84 (0.23)	1.01 (0.92, 1.10)
Non-HDL cholesterol (per + 30 mg/dl)	813	123.1 (31.2)	141.2 (39.4)	0.74 (0.60, 0.90)
HDL cholesterol (per + 10 mg/dl)	813	52.1 (14.5)	53.7 (16.0)	0.93 (0.81, 1.08)
hsCRP (mg/L)	803	3.2 (7.0)	3.3 (6.2)	1.05 (0.83, 1.33)^d^
IL-6 (pg/ml)	803	3.0 (4.4)	2.5 (5.0)	1.28 (1.02, 1.62)^d^

Incident Brain Aging was defined as having a principal component analysis (PCA) score ≥−1 at baseline and <−1 at follow-up; there were *n* = 73 with incident brain aging and *n* = 749 without brain aging at this study's 5-year follow-up (EHLS 20-year follow-up: *n* = 822); EHLS, epidemiology of hearing loss study; BMI, body mass index; RR, relative risk; CI, confidence interval; HDL, high density lipoprotein; hsCRP, high sensitivity C-reactive protein; IL-6, interleukin-6; IMT, carotid artery intima media thickness.

^a^ Results of Poisson models with each individual baseline risk factor as determinant and incident brain aging as outcome adjusted for age and sex.

^b^ Adjusted for age only.

^c^ Adjusted for sex only.

^d^ Natural log transformed (ln).

In age-sex-adjusted models, a history of stroke, obesity, depressive symptoms and elevated non-HDL cholesterol and IL-6 were associated with an increased risk for brain aging 5 years later. Additionally, in the secondary analyses, less years of education and arterial stiffness were associated with a higher risk of brain aging; obesity and non-HDL cholesterol were not significant in the secondary analyses. The small number of incident cases of brain aging did not allow for sex-specific analyses.

In the age-adjusted multivariable model, sex (RR = 1.62, 95% CI = 1.03, 2.53, men), a history of stroke (RR = 2.89, 95% CI = 1.71, 4.88), and obesity (RR = 1.71, 95% CI = 1.07, 2.73, BMI > 30) were associated with the 5-year incidence of brain aging. Less years of education (RR = 0.48, 95% CI = 0.25, 0.93, 16 + years) and lower non-HDL cholesterol (RR = 0.75, 95% CI = 0.60, 0.93, per 30 mg/L increase) were associated with an increased risk for developing brain aging (Table 4, Model 1). The estimate of IL-6 did not remain statistically significant when modelled with stroke and was not retained. Arterial stiffness (RR = 1.55, 95% CI = 1.03, 2.33) was associated with an increased risk for brain aging in the secondary analyses (Table 4, Model 2).

**Table 4:** Multivariable models of risk factors and 5-year incidence of brain aging.

	Model 1^a^	Model 2^b^
Baseline risk factors	RR (95% CI)	RR (95% CI)
Age (per + 5 years)	1.91 (1.64, 2.22)	1.77 (1.55, 2.02)
Sex (Male)	1.62 (1.03, 2.53)	1.58 (1.08, 2.30)
Education (≥16 years)	0.48 (0.25, 0.93)	0.46 (0.26, 0.82)
Stroke	2.89 (1.71, 4.88)	2.31 (1.31, 4.07)
Obesity (BMI > 30 kg/m^2^)	1.71 (1.07, 2.73)	1.38 (0.94, 2.04)
Non-HDL cholesterol (per +30 mg/L)	0.75 (0.60, 0.93)	0.91 (0.77, 1.06)
Arterial stiffness	1.17 (0.73, 1.89)	1.55 (1.03, 2.33)

CI, confidence interval; RR, relative risk; BMI, body mass index; HDL, high density lipoprotein.

^a^ Results of the multivariable Poisson model with baseline risk factors as determinants and incident brain aging as outcome; *N* = 715.

^b^ Results of the multivariable Poisson model with baseline risk factors as determinants and incident brain aging as outcome, including participants with cognitive impairment/dementia without a PCA score in the brain aging group; *N* = 739 (secondary analyses).

## Discussion

We found that risk factors of lifestyle, vascular and metabolic health and inflammation were associated with sensorineural and neurocognitive function (brain aging) in older adults. Participants with brain aging were more likely to have fewer years of education, not drink alcohol or to have a history of heavy drinking. Obesity was associated with the development of brain aging. A history of stroke and arterial stiffness, both markers of advanced atherosclerosis were associated with the development of brain aging and depressive symptoms with prevalent brain aging. Moreover, blood measures of inflammation and non-HDL cholesterol, a well-known cardiovascular risk factor, were identified as determinants of brain aging. These results add to existing evidence that lifestyle, vascular and metabolic health, and inflammatory factors could contribute to brain aging in older adults. We extend previous studies by the study of a large, well-characterized cohort of older adults. Furthermore, we utilized objective assessments of sensorineural and neurocognitive function to capture brain aging, which helps to determine estimate effects of risk factors on a more holistic composite of brain function.

In our study, a number of factors of lifestyle, vascular and metabolic health and inflammation were associated with brain aging in age-sex adjusted models, while not all associations remained in multivariable models. Effect sizes from significant effects in the multivariable models ranged from ∼50% increased risk to 2.5-fold increase risks for dichotomous risk factors.

Fewer years of education were associated with more brain aging. Individuals with less than 16 years for education were approximately 50% more likely to have prevalent or incident brain aging as compared to those with 16 years of education or more. This is consistent with research showing a protective effect of more education on cognition and sensory and brain health (4, 27, 30, 45, 46).

We also found more brain aging in individuals who did not drink alcohol and those who had a history of heavy drinking. The association of neurodegeneration with alcohol consumption in humans is likely complicated and not linear. While heavy drinking and alcohol use disorders are considered risk factors for dementia (47, 48) and might influence an earlier development through neurotoxicity, thiamine deficiency, other conditions, and/or vascular pathways (47), there are also studies reporting neuroprotective effects of smaller doses of alcohol intake (49), and other studies show an increased risk in people who are abstinent (50). Similar inconsistencies have been found with regards to alcohol consumption and hearing loss (51–54). A longitudinal study of drinking and cognitive performance showed a nonlinear association between drinking patterns and later life cognitive performance, where nondrinkers and heavy drinkers had worst cognitive results (55). The disadvantageous effects in individuals who are not drinkers might be because such individuals might have comorbidities and other underlying diseases or be taking medications, that may not allow them to drink.

We also found more brain aging in obese individuals. Overweight and obesity have been shown to be risk factor for cognitive decline and hearing loss (52, 56–60). Harmful effects of obesity on the brain might be due to metabolic dysregulation (e.g., elevated lipids and/or insulin resistance) and systemic inflammation (61).

In our study, we also found that higher levels of IL-6, a blood measure of inflammation was associated with more brain aging. Chronically elevated levels of circulating pro-inflammatory markers have been observed with aging and are associated with symptoms of chronic disease (62) and elevated inflammation has been reported to be associated with cognitive decline, dementia, and neuronal health (20, 63–66).

In contrast, the associations of cholesterol blood measures with brain health are likely complex and studies have shown varying results. Higher cholesterol levels in midlife have been considered a risk factor for Alzheimer's disease and dementia (67, 68). However, in older populations a higher non-HDL cholesterol may represent better health as lower values have been associated with frailty, disease, and an increased risk for mortality (69). In our study of older adults, a higher non-HDL cholesterol was associated with a reduced risk of developing brain aging based on the PCA score. There was, however, no effect in the secondary model, where individuals with cognitive impairment/dementia and missing PCA data had been included in the brain aging group.

We found risk factors for chronic vascular diseases (stroke and atherosclerosis) to be associated with brain aging, which is in line with existing research on sensory and cognitive outcomes (2, 13, 22, 70, 71).

Depressive symptoms were a risk factor for brain aging in our study. There could be multiple mechanisms underlying this association; depressive symptoms may occur as a result of pathophysiological brain changes that occur with age or disease or, be in response to perceived decline in function, and there could be a differential effect related to age or health status (72).

Many, but not all, factors associated with brain aging in this study were similar to those reported in the younger BOSS cohort (18). In both studies, education, lifestyle factors as well as factors of vascular and metabolic health and inflammation were identified as risk factors for brain aging. The exact conditions were not always the same in both studies. For instance, there was an association of history of heavy alcohol consumption in this study in the older EHLS cohort, whereas, there was an association of more exercise with brain aging in the younger BOSS cohort. This is in agreement with research suggesting that risk factors for brain aging and neurodegeneration may differ in their impact across the life span (73–75).

The brain is a vital component of the sensorineural systems and responsible for the perception, interpretation, and response to sensorineural signals coming from the sensory organs. Therefore, declines in brain function may be reflected in changes of sensorineural function similar to declines in neurocognitive function. In this study, we used a composite measure of sensorineural and neurocognitive function as a marker of brain aging (18). This expanded measure of brain function accounts for the perception and processing of sensory stimuli in addition to the cognitive function domains of memory, executive function, processing speed and language. This fills in gaps of previous literature, focusing on the association of senses as a determinant and cognition as an outcome (5, 7–12). Moreover, we were able to determine the effect of numerous risk factors of lifestyle, vascular and metabolic, health and inflammation in a global measure of brain health adding to previous work on individual sensory or cognitive outcomes (2, 5, 7, 13–17, 19–27). The consistent findings across the EHLS and BOSS suggest that at older ages, as well as in midlife, lifestyle factors, vascular and metabolic health and inflammation were associated with poorer scores on neurological function. Importantly, many of these factors are considered modifiable and could, thus, present an opportunity to inform future intervention and prevention strategies to promote healthy brain aging. Due to this idea of a shared etiology, such treatments would then likely simultaneously benefit a multitude of functional outcomes, including sensory and cognitive function.

### Limitations and strengths

We had missing cognitive test data in participants with MCI/dementia but were able to classify them based on previous diagnoses as cases of brain aging. Most of the results were similar in these secondary analyses. Because our sensorineural functional measures evaluated the whole sensorineural system, it was not possible to separate out potential contributions of peripheral organ dysfunction to the brain aging marker. Residual confounding may have limited our ability to detect some factors associated with brain aging. The EHLS is predominantly non-Hispanic White, which may limit our ability to generalize our findings to other populations. More research in more diverse cohorts will be needed to determine which factors may play the biggest role for brain aging at which time points in life.

The strengths of our study are the large, well-characterized prospective cohort study with ages ranging from the mid-60's into the 90's. We were able to investigate a variety of lifestyle, inflammation, vascular, and metabolic health factors using objective physical examination data and standardized lab procedures. We were utilizing repeated measures of objectively assessed sensorineural and neurocognitive function in an incidence design. Additionally, results in the current study were consistent with those reported previously in the middle-aged BOSS cohort (18).

## Conclusion

We identified several factors associated with brain aging in older adults. Our results add to the evidence of shared vascular, metabolic and inflammatory etiologic pathways for sensorineural and neurocognitive declines associated with aging. Moreover, some of the risk factors are considered modifiable giving rise to future research on possible intervention and prevention strategies. Targeting the shared central brain processing etiological factors with interventions may lead to retention of better neurological function impacting multiple systems, including sensory and cognitive function and might thus help older adults retain their independence and a better quality of life longer.

## Data Availability

The datasets presented in this article are not publicly available because of data protection regulations. Requests to access the datasets through data sharing agreements should be directed to NM; natascha.merten@wisc.edu.
