# Supplementary material for: Lifestyle and factors of vascular and metabolic health and inflammation are associated with sensorineural-neurocognitive aging in older adults
Source: Front Epidemiol. 2024 Jan 5;3:1299587. doi: 10.3389/fepid.2023.1299587 (PMC10910988; doi:10.3389/fepid.2023.1299587)
Supplement: Supplementary file 1 [file Table1.docx]

**Supplementary Materials**

**Lifestyle and Factors of Vascular and Metabolic Health and Inflammation are Associated with Sensorineural-Neurocognitive Aging in Older Adults**

Natascha Merten, PhD, MS, Mary E Fischer, PhD, A. Alex Pinto, MS, Richard J. Chappell, PhD, & Carla R Schubert, MS

Supplementary Material 1. Principal Components Analysis (PCA) with Baseline Sensorineural and Neurocognitive Measures.

Supplementary Material 2. Mean Sensorineural and Neurocognitive Function Test Measures at Baseline & 5-year Follow-ups by Prevalent and Incident Brain Aging.

**Supplementary Material 1. Principal Components Analysis (PCA) with Baseline Sensorineural and Neurocognitive Measures**

| **Measure** | **Factor Loadings** | **% Variance Explained ^a^** |
| --- | --- | --- |
| Pure-tone Threshold Average at 0.5, 1, 2, 4 kHz^b^ | 0.45 | 20.2 |
| San Diego Odor Identification | 0.57 | 32.3 |
| Trail Making Test, Part A^b^ | 0.77 | 58.9 |
| Trail Making Test, Part B^b^ | 0.82 | 67.3 |
| Auditory Visual Learning Test | 0.64 | 40.4 |
| Digit Symbol Substitution Test | 0.83 | 69.5 |
| Verbal Fluency Test | 0.56 | 31.5 |
| Mini-Mental State Examination | 0.68 | 46.4 |

Note: kHz, kilohertz. Data of this study baseline was from the 15-year follow-up of the Epidemiology of Hearing Loss Study (EHLS).

^a^ Based on PCA commonalities

^b^ Data entered as additive inverses so that higher numbers indicated better function

**Supplementary Material 2. Mean Sensorineural and Neurocognitive Function Test Measures at Baseline & 5-year Follow-ups by Prevalent and Incident Brain Aging.**

|  | **Prevalent Brain Aging** | | **5-year Incident Brain Aging** | |
| --- | --- | --- | --- | --- |
|  | Yes (n=208) | No (n=1270) | Yes (n=73) | No (n=749) |
| **Function Measure** | **Mean (Range)** | **Mean (Range)** | **Mean (Range)** | **Mean (Range)** |
| PTA at 0.5, 1, 2, 4 kHz (dBHL) worse ear | 48.2 (8.8-125) | 31.5 (2.5-125) | 52.6 (6.3-123) | 34.7 (5.0-125) |
| San Diego Odor Identification (# odors) | 4.1 (0-8) | 6.6 (0-8) | 4.6 (1-8) | 6.6 (0-8) |
| Trail Making Test, Part A (seconds) | 75.6 (32-259) | 38.9 (14-109) | 67.5 (31-146) | 39.7 (18-105) |
| Trail Making Test, Part B (seconds)^a^ | 231.7 (105-301) | 107.4 (35-301) | 229 (124-301) | 108.2 (41-301) |
| Auditory Verbal Learning Test (# words) | 2.8 (0-8) | 5.6 (0-15) | 2.9 (0-8) | 5.7 (0-14) |
| DSST (# symbols) | 26.4 (4-46) | 44.0 (19-75) | 27.8 (13-47) | 43.7 (22-80) |
| Verbal Fluency Test (# words) | 23.9 (3-54) | 34.9 (6-79) | 26.8 (7-45) | 36.0 (8-74) |
| Mini-Mental State Examination Score | 25.5 (7-30) | 28.5 (23-30) | 26.7 (23-30) | 28.7 (23-30) |

Note. Participants with brain aging have PCA standardized score > 1 standard deviation below the mean PCA (<-1). PTA, Pure-tone Average; kHz, kilohertz; dB HL, decibel hearing level; DSST, Digit Symbol Substitution Test. Data of this study comes from the 15-year follow-up (baseline) and 20-year follow-up of the Epidemiology of Hearing Loss Study (EHLS).

^a^ A score of 301 was assigned to participants who were unable to complete the test in 300 seconds.
